# Social media analysis reveals environmental injustices in Philadelphia urban parks

**DOI:** 10.1038/s41598-023-39579-4

**Published:** 2023-08-03

**Authors:** Matthew Walter, Benjamin E. Bagozzi, Idowu Ajibade, Pinki Mondal

**Affiliations:** 1 Department of Geography and Spatial Sciences, University of Delaware, Newark, DE 19716 USA; 2 Department of Political Science and International Relations, University of Delaware, Newark, DE 19716 USA; 3 Department of Environmental Sciences, Emory University, Atlanta, GA 30322 USA; 4 Department of Plant and Soil Sciences, University of Delaware, Newark, DE 19716 USA

**Keywords:** Environmental impact, Sustainability, Socioeconomic scenarios, Urban ecology

## Abstract

The United Nations Sustainable Development Goal (SDG) target 11.7 calls for access to safe and inclusive green spaces for all communities. Yet, historical residential segregation in the USA has resulted in poor quality urban parks near neighborhoods with primarily disadvantaged socioeconomic status groups, and an extensive park system that addresses the needs of primarily White middle-class residents. Here we center the voices of historically marginalized urban residents by using Natural Language Processing and Geographic Information Science to analyze a large dataset (n = 143,913) of Google Map reviews from 2011 to 2022 across 285 parks in the City of Philadelphia, USA. We find that parks in neighborhoods with a high number of residents from historically disadvantaged demographic groups are likely to receive lower scores on Google Maps. Physical characteristics of these parks based on aerial and satellite images and ancillary data corroborate the public perception of park quality. Topic modeling of park reviews reveal that the diverse environmental justice needs of historically marginalized communities must be met to reduce the uneven park quality—a goal in line with achieving SDG 11 by 2030.

## Introduction

Racial and ethnic minorities and people with disadvantaged socioeconomic status (SES) often have limited or no access to high quality urban parks^1–6^. This challenge has persisted for decades prompting the focus on safe, inclusive and accessible urban green space (UGS) for the Sustainable Development Goal (SDG) 11 target 7^7^, especially considering women, children, older persons, and persons with disabilities. Environmental justice requires the assurance that all communities have the right to live, work, and play in a healthy and safe environment, and that the burdens of environmental harms are minimized while benefits such as access to green spaces are distributed equitably^3,8^. Yet, institutional practices, such as residential segregation including redlining and blockbusting^9,10^, have historically led to an environmental injustice in the distribution of high quality urban parks in the USA.

Redlining was a discriminatory practice first used by the Home Owners’ Loan Corporation (HOLC) in the 1930s to classify areas with predominantly Black, immigrants, and low-income residents as “hazardous” or high-risk to mortgage lenders, thus disenfranchising these groups from accessing loans. Consequently, this facilitated neighborhood disinvestment, lower geographic mobility, reduced homeownership, and inferior home quality^11,12^. Although redlining was outlawed in 1968, four years after the Civil Rights Acts of 1964 prohibited racial segregation, its legacies are still felt today, with once redlined neighborhoods experiencing worse health outcomes, higher heat indexes, and less green space^13–15^. Blockbusting was another tactic leading to segregation in which real estate managers would pressure White homeowners to sell their homes cheaply due to the fabricated fear of other races and economic classes moving into the neighborhood^16^. These discriminatory practices caused city centers with less park space to be disinvested and increasingly composed of racial minorities, while White populations migrated to suburban areas^17^.

Modern day efforts to increase UGS and improve related amenities are often unable to reverse historical inequality, and in some cases, have led to green gentrification, i.e., the displacement of demographic groups because of increased housing prices that resulted from urban greening^18,19^. One such example is the creation of the High Line, a park built on an abandoned rail line in Manhattan during 2009–2014, which increased housing costs by 35% in properties closest to the park^20^. Persistent drivers of inequality are continuously being identified, such as decreased park spending in areas with high poverty and Black and Asian populations^21–23^.

There is no dearth of research on environmental injustice related to UGS^24–26^. Yet, most distributive justice research on park quality utilizes external metrics and tools created by researchers to assess park quality which do not always take into account cultural differences in park preference^27^. This may be problematic when assessing the quality of USA urban parks which have been historically designed to serve the needs of White upper-class residents^1^. To ensure the opinions and expectations of historically neglected residents can be incorporated into future park planning, it is critical to focus on the intersections of environmental justice with other forms of justices such as interactional, distributive, and spatial justice. Interactional justice refers to the consideration of the dignity and needs of people being impacted by a policy decision. Distributive and spatial justice addresses issues of equity and fairness in distribution of burdens, resources, and social goods that promote safety, well-being and health in different geographic spaces including neighborhoods^28^. Spatial justice specifically considers the interconnectedness between urban development, spatial arrangements, and social justice. It seeks to create more sustainable and inclusive landscapes that benefit all members of society regardless of race, socio-economic status, abilities, geographic location, or other qualities. Here, we examine the intersections of these justice issues in Philadelphia by measuring the perception of park quality directly from park users’ social media reviews and performing a spatial accessibility analysis between perceived park quality and residential demographics. We gain further insight from the social media reviews by using topic modeling to assess the drivers of park quality perception and comparing these drivers to observed park data.

Examining equitable UGS is critical for Philadelphia as it ranks high among USA cities in metrics of racial and SES inequality. In 2017, Philadelphia had a poverty rate of 25.7% for 1.57 million residents, more than double the national average, and the highest rate of deep poverty (income 50% below the poverty line) amongst all large USA cities^29,30^. The poverty rate for Hispanic and Black residents in Philadelphia was even higher in 2017 compared to that of the overall city population, with 37.9% and 30.8% living below the poverty line respectively. Philadelphia is home to an extensive urban park system, with Philadelphia Parks and Recreation managing more than 300 urban parks with 267 km of trails, more than 600 baseball, football, and soccer fields, and more than 600 basketball and tennis courts. Philadelphia parks are also highly accessible, with 95% of the population living within a 10-min walk to a park compared to the national average of 55%^31^. However, annual park spending per capita is comparatively low in Philadelphia at US $73/person compared to the national average of US $98/person in 2022^31^. Moreover, those living in neighborhoods of color have 28% less park space than those in White neighborhoods, whereas low-income neighborhoods have 42% less park space than high-income neighborhoods^31^. In order to stay on track with SDG 11, it is crucial for any near-term urban planning endeavors to account for public perceptions of the existing park systems, and demographic group-specific preferences.

We used a combination of cutting-edge methods of Natural Language Processing (NLP) and Geographic Information Science (GIS) to determine urban park quality in Philadelphia, with a specific focus on demographic-specific needs. First, we performed an origin and destination (OD) cost matrix analysis^32^ to determine whether different demographic groups have disproportionate access to parks of different qualities (see “Methods”). 21 demographic variables representing various racial/ethnic, SES, and other demographic groups were selected from the American Community Survey (ACS) 5-year estimates in six categories: race and ethnicity, age, sex, SES (education, income, unemployment, food assistance, and health insurance), people with disabilities, and language. We then analyzed 143,913 reviews from 2011 to 2022 for 285 parks that we scraped from Google Maps to understand the perception-based quality of parks. The text-based content of user reviews was analyzed using anchor word-assisted topic modeling to determine what park characteristics were commonly cited in positive and negative reviews for parks within neighborhoods composed of different proportions of racial/ethnic, SES, gendered, age, and disability groups (see Methods). Next, we evaluated these parks for their physical characteristics by developing land cover maps from high-resolution aerial (1 m) and moderate resolution satellite images (10 m) along with ancillary park data reported by Philadelphia Parks and Recreation. Finally, we assessed the association between user perceptions and physical characteristics of parks. Our objective is to answer the following questions: (1) Do certain neighborhoods and demographic groups have disproportionate access to high quality parks? (2) What perceived park characteristics lead to a positive or negative experience? (3) Do these user perceptions align with park characteristics captured with remotely sensed images and ground data? We present the results of our findings below.

## Results

### Perceived park quality reveals distributive injustice

**Figure 1:**
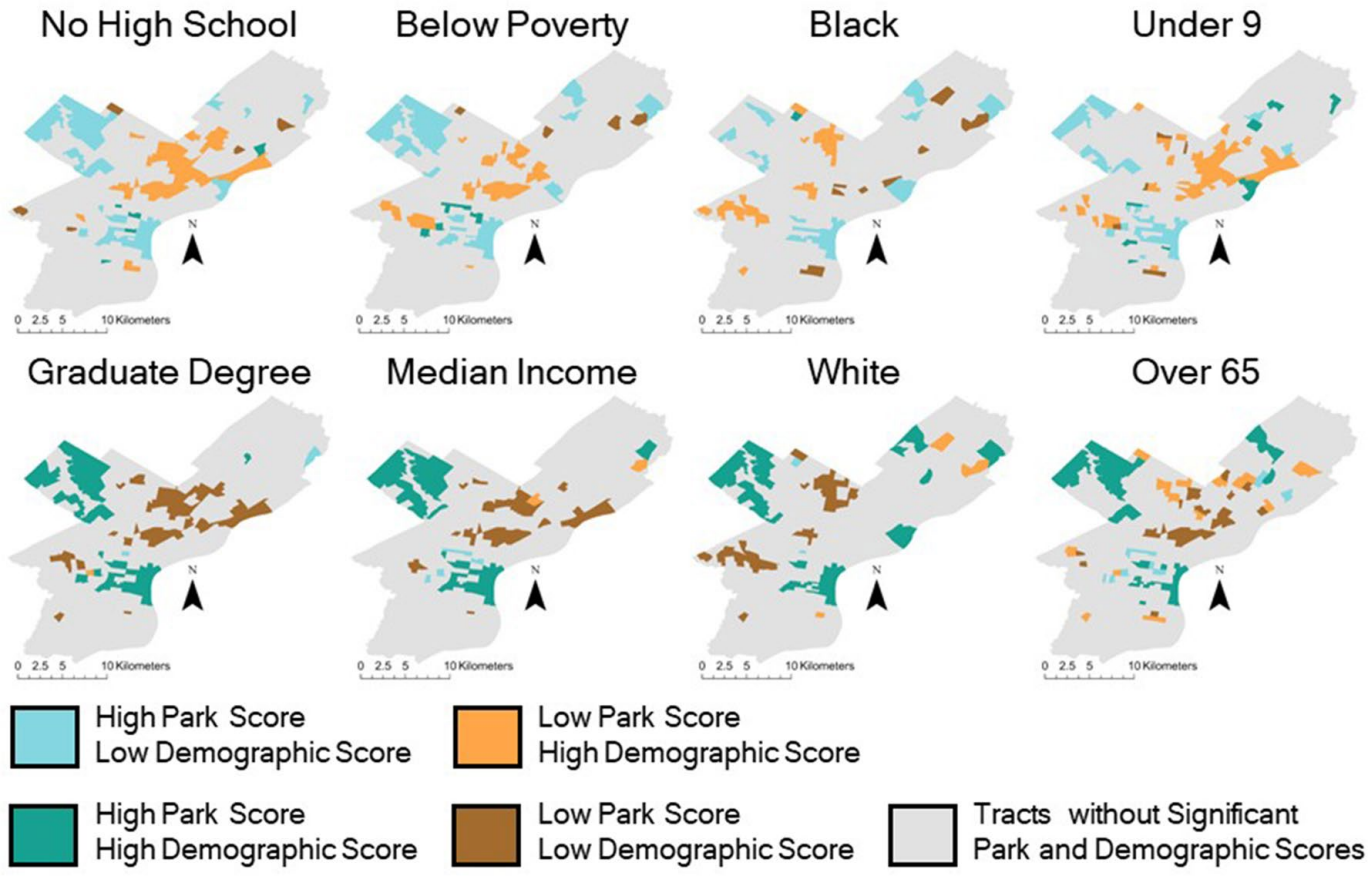
Relationships between three demographic groups (education, income, and race) and their park score at the census tract level, measured as the average Google Map score of all parks within 800 m of a tract’s residential areas. These bivariate maps can be used to identify areas in need of park improvements due to having low park scores and high concentrations of certain demographic groups. A complete set of bivariate maps for all the significant demographics included in this study can be found in Supplementary Information Fig. 1.

**Figure 2:**
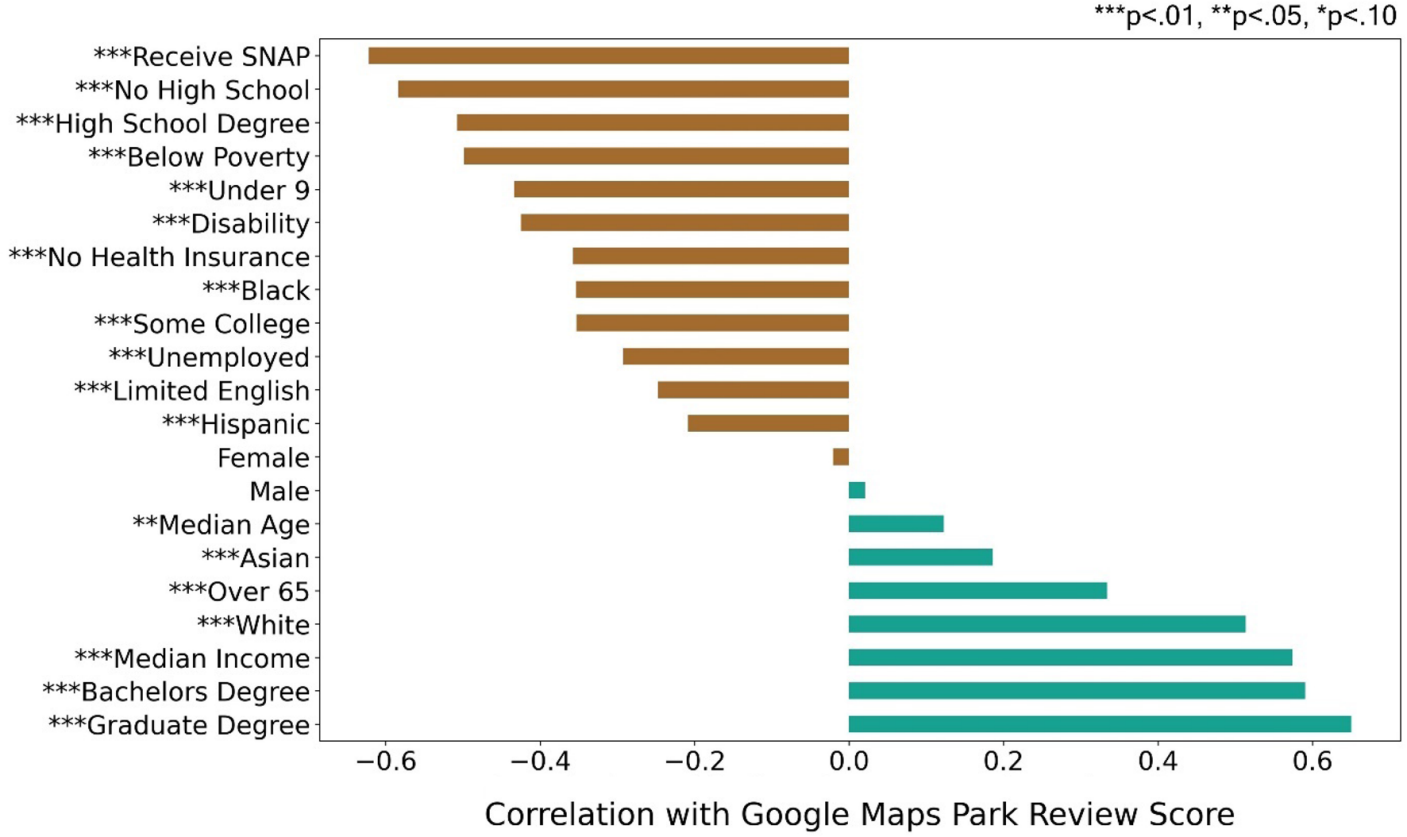
Correlation coefficients between socioeconomic variables and park score determine which variables are positively or negatively associated with park scores and the strength of those relationships. P-values of the correlation coefficients are shown as measures of statistical significance, with * representing a p-value of less than 0.1, ** a p-value of less than .05, and *** a p-value of less than 0.01.

We found a clear pattern of parks with lower scores being consistently associated with predominantly Black and disadvantaged SES neighborhoods (Figs. 1 and 2). Young children, people who are living below the poverty level, without high school or college degrees, with a disability, who receive aid from the Supplemental Nutrition Assistance Program (SNAP), unemployed, without health insurance, and Black populations all tend to live in census tracts with lower park scores (Fig. 1). Census tracts with high proportions of SNAP recipients and people with no high school degree have average park scores that are 0.33 and 0.32 stars (on a scale of 1–5 stars; see “Methods”) lower respectively than tracts with low proportions of these demographic groups.

We also found that people over 65, with higher median incomes, with a college degree, and White populations tend to live in areas with higher park scores (Fig. 1), indicating that these groups have disproportionate access to higher quality parks. Census tracts with higher proportions of higher-income, bachelor’s degree, and graduate degree holding residents receive more favorable park ratings, an increase of 0.31, 0.33, and 0.37 stars respectively, over tracts with low proportions of these demographic groups (Fig. 2). This is a substantively sizable effect given that the sample standard deviation on park scores itself is 0.30 stars. Areas with higher White and advantaged SES populations and higher park scores are located in Northwest and Central Philadelphia (Fig. 1), that host several highly visited and rated parks including Wissahickon Valley Park, Love Park, Rittenhouse Square, Logan Square, Washington Square, and Franklin Square.

### Drivers of public park quality perception

**Figure 3:**
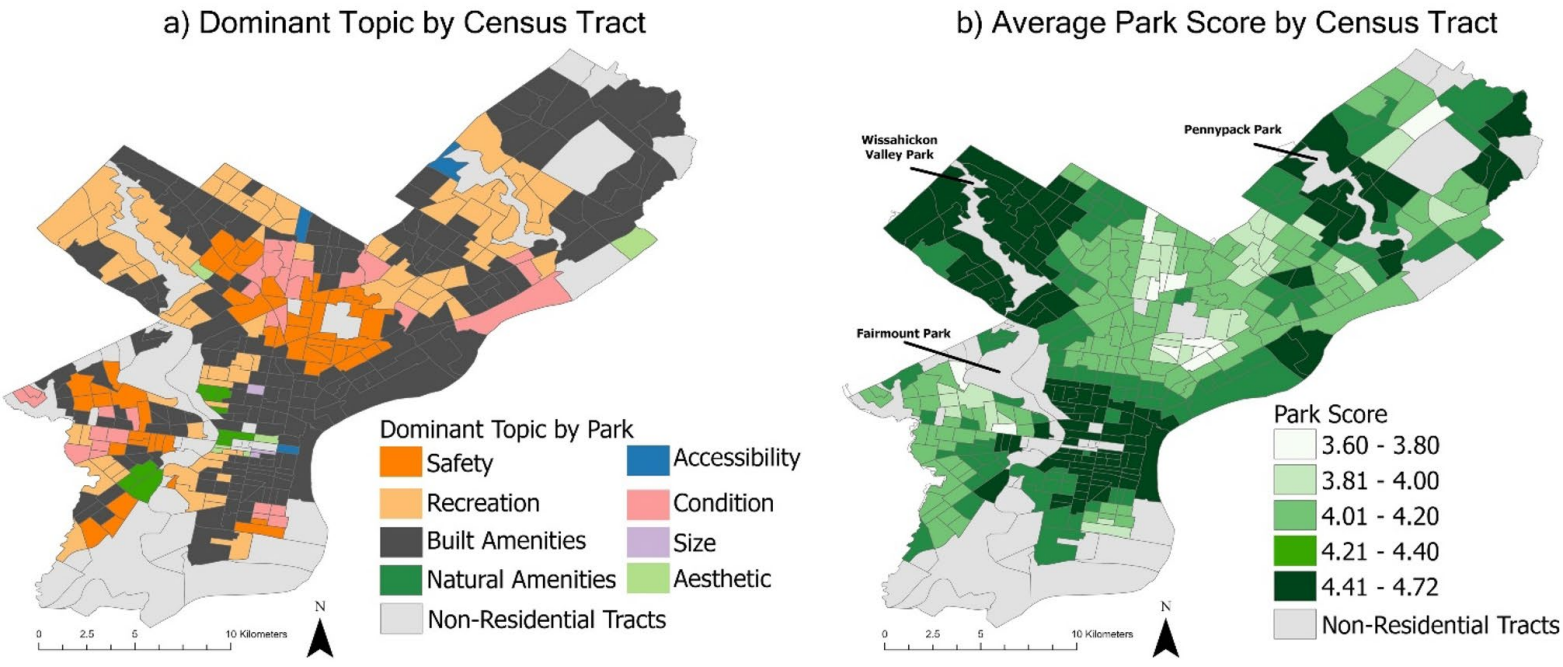
Eight review topics representing common park features that influence overall park score. (**a**) Displays the topic most likely to be mentioned by users in all of the parks within 800 m of a census tract. These dominant topics for each census tract can be compared to (**b**) that displays the average park score based on the number of stars given by users to all parks within 800 m of a census tract.

To evaluate the reasoning behind positive and negative park reviews left on Google Maps, we utilized the text-based portion associated with the reviews (n = 69,686; see “Methods”). The dominant topic most common throughout the city is ‘built amenities’, followed by ‘recreation’ and ‘safety’ (Fig. 3a). Tracts with high park scores cluster around two large nature-based parks, Wissahickon Valley Park and Pennypack Park, where the dominant topic is ‘recreation’ (Fig. 3b). A third cluster of high park scores in Center City has a higher heterogeneity in dominant topics, including ‘built amenities’, ‘recreation’, ‘natural amenities’, ‘accessibility’, ‘size’, and ‘aesthetic’. There are two clusters of tracts with low park scores in North and West Philadelphia that predominantly have ‘safety’ and ‘condition’ as their dominant topics.

**Figure 4:**
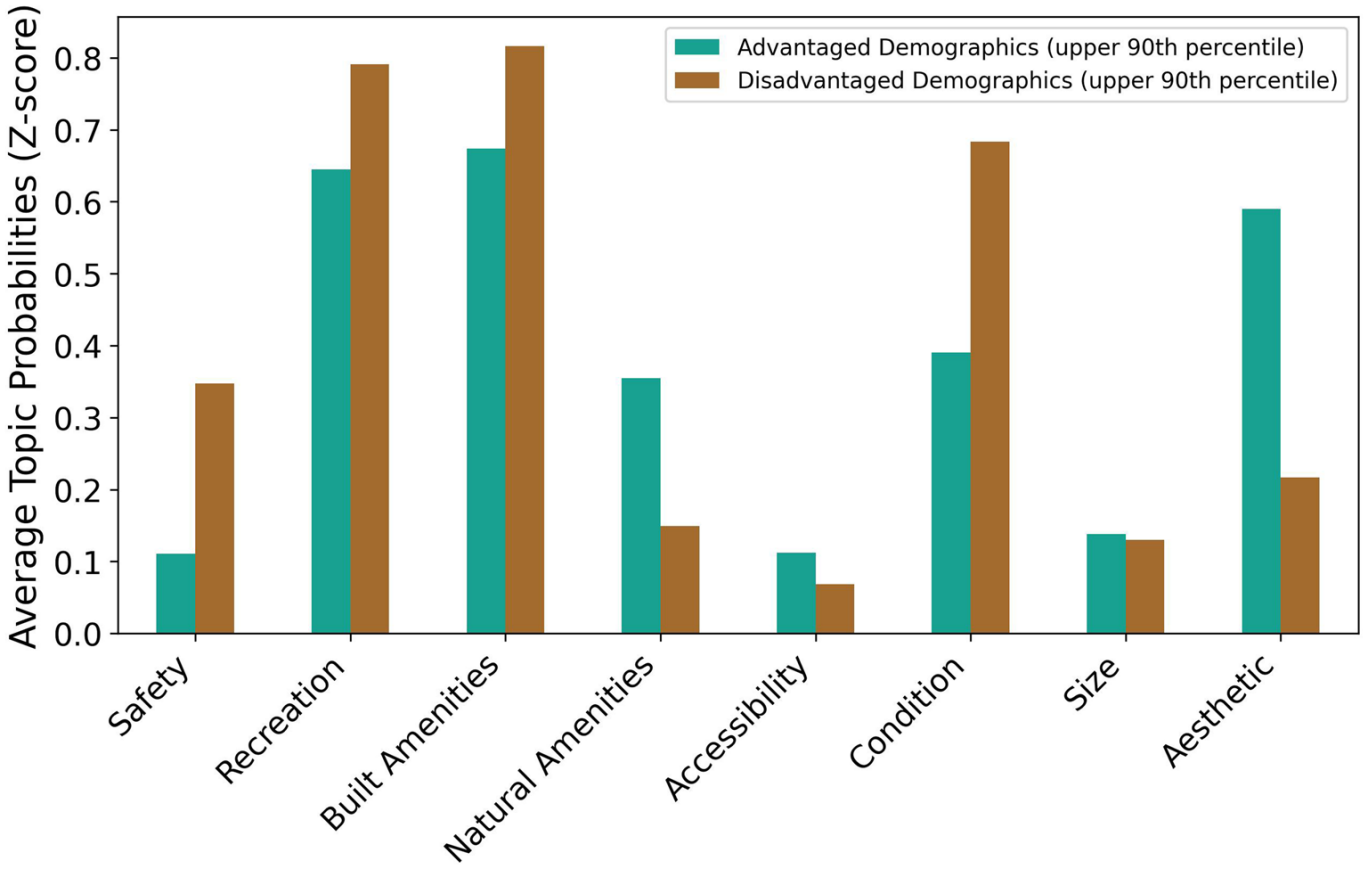
The probability that a topic is mentioned in reviews of parks that are accessible to census tracts with both advantaged and disadvantaged demographic compositions. Advantaged demographics tracts are tracts in which demographics positively correlated to park score (over 65, White, median income, bachelor’s degree, and graduate degree) are in the upper 90th percentile (n = 99). Disadvantaged demographics tracts are tracts in which demographics negatively correlated to park score (receive supplemental nutritional assistance, less than a high school degree, high school degree, under 9, disability, no health insurance, Black, and some college) are in the upper 90th percentile (n = 175).

We found that topics associated with positive reviews (‘aesthetic’ and ‘natural amenities’; Supplementary Information Fig. 2) are more likely to be mentioned in reviews of parks that are accessible to census tracts with predominantly advantaged populations including White, high-income, and higher educated residents, while topics associated with negative reviews (‘safety’ and ‘condition’; Supplementary Information Fig. 2) are more likely to be mentioned in reviews of parks that are accessible to census tracts with predominantly disadvantaged populations including Black and Hispanic residents, disadvantaged SES groups, children, and those with disabilities (Fig. 4).

**Figure 5:**
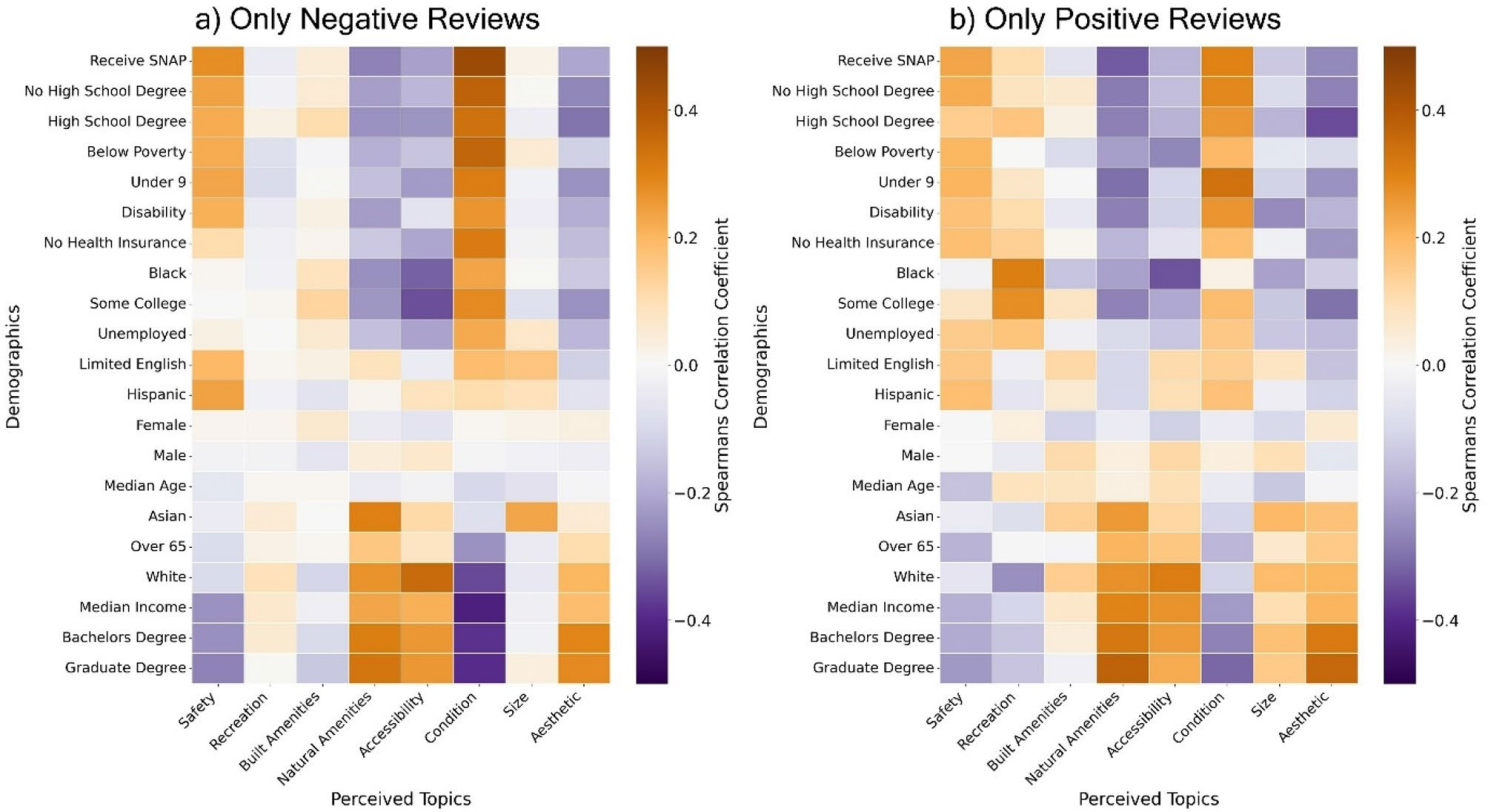
Heat maps showing the relationships between all of the demographic variables in a census tract and how likely a topic is to be mentioned by parks accessible to that census tract. Panels show how different demographic groups are mentioning a review topic in (**a**) a negative review or (**b**) a positive review.

Comparisons of correlations between specific demographic groups and topic probabilities reveal additional differences in perception of park quality (Fig. 5a, b). For example, negative reviews of parks near predominantly Black neighborhoods are more likely to mention park condition while those near Hispanic neighborhoods are more likely to mention safety, and those near White neighborhoods are more likely to mention accessibility. Positive and negative reviews of parks in senior-dominated areas are more likely to mention natural amenities, while areas with more children are more likely to mention condition and safety when compared to other topics. Reviews in higher-income areas are more likely to highlight natural amenities and accessibility as park qualities, while reviews in higher poverty areas and areas of lower educational attainments are more likely to have concerns of safety and condition. Similarly, disadvantaged SES groups including those who receive SNAP, who have no health insurance, and who are unemployed are all more likely to live near parks exhibiting higher levels of concern over park condition and safety.

### Evidence linking public perception to observed park qualities

In order to compare perception to observation, we measured nine different variables for each park, based on data obtained from the Philadelphia Police Department, Philadelphia Parks and Recreation, and aerial/satellite imagery. These observed park characteristics reflect six out of the eight topics from the previous section: ‘safety’, ‘recreation’, ‘built amenities’, ‘natural amenities’, ‘size’, and ‘aesthetic’ (Supplementary Information Fig. 3). Ancillary data were not available to represent ‘condition’ or ‘accessibility’.

**Figure 6:**
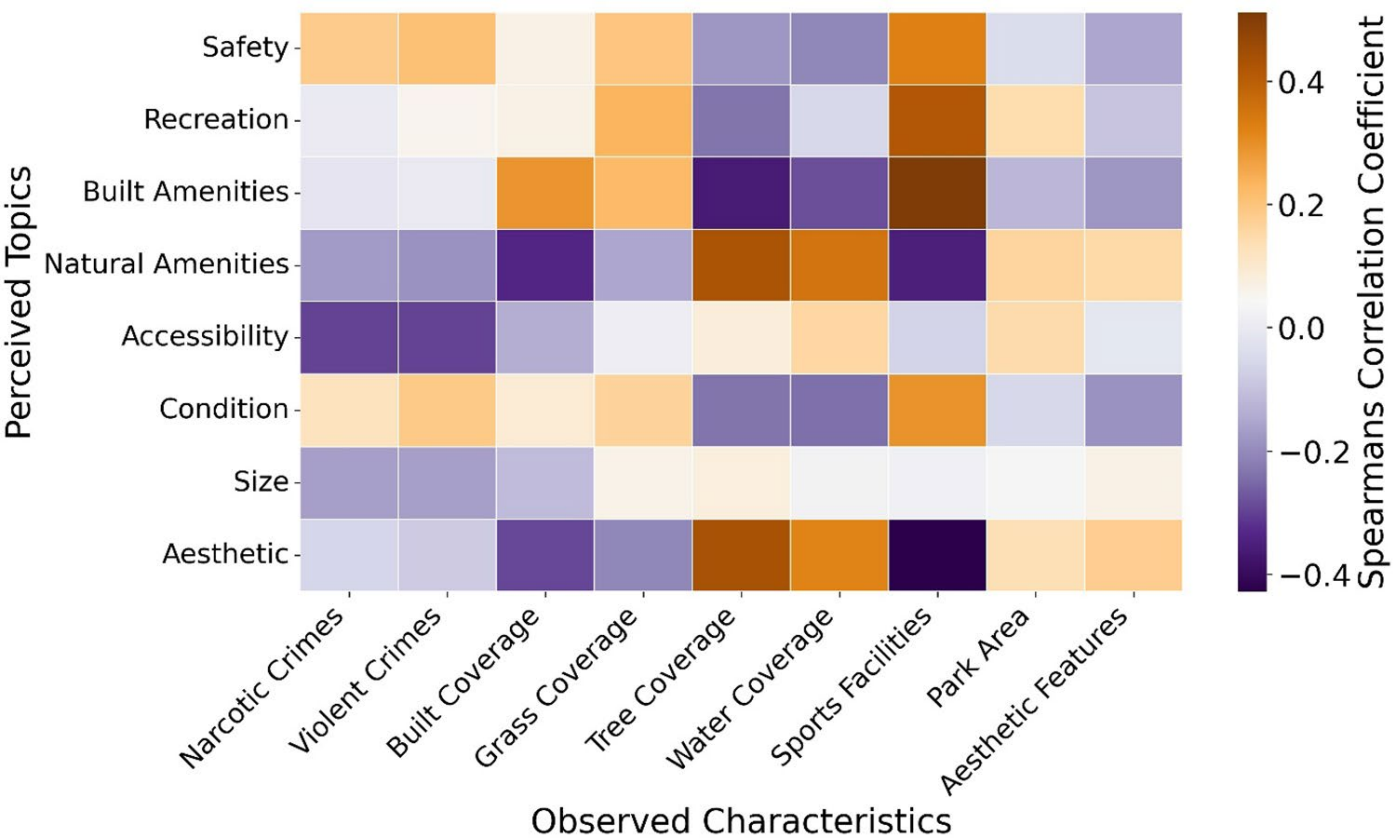
A heatmap of correlation coefficients between the likelihood of each of the eight perceived topics being mentioned for a park and the occurrence of each observed variable within a park.

We found a statistically significant relationship between a park’s perceived topics and observed characteristics using Spearman’s Rank Correlation, but only in some cases (Fig. 6). Positive associations exist between ‘recreation’, ‘natural amenities’, ‘built amenities’, and ‘aesthetic’ and at least one of their respective observed characteristics. Such associations indicate that user reviews of parks on Google Maps can accurately and objectively identify observed characteristics of a park. For example, the ‘natural amenities’ and ‘aesthetic’ topics positively correlate with the amount of tree and water coverage in a park. This result, coupled with the results from the previous section, indicates that the perception of natural amenities and aesthetics in a park visit, which were associated with a higher park score, can be improved by increasing the tree and water coverage within a park.

The safety topic correlates only weakly with the observed incidence of violent and narcotic crimes while unexpectedly correlating more strongly with the number of sports facilities. The park size topic also does not correlate with the overall size of a park or any of the other observed park characteristics (Fig. 6 and Supplementary Information Fig. 3). This indicates that some perceived park topics may not directly connect to their real-world metrics and are instead influenced by a variety of external factors that influence one’s feelings of safety such as lighting, proximity to vehicle traffic, and unsafe recreational equipment. This complexity makes identifying solutions for improving safety less straightforward.

## Discussion

Prior research has indicated that park characteristics viewed favorably by one demographic group are not always viewed in the same way by other groups^1,33^. White Americans are more likely to visit parks to engage in camping, hiking, hunting, boating, swimming, cycling, and dog-walking with a focus on seclusion and individualism, whereas Black Americans are more likely to participate in social, formal, and sports-oriented activities when visiting parks^1^. Women are more likely to use parks with improved aesthetics compared to men, while children are more likely to use parks with increased activity settings such as swing sets, basketball courts, and sports fields^33^. This study measures park quality using an approach based on a large dataset of social media reviews that accounts for differences in user preferences by aggregating opinions on park quality directly from those who are using the park. Our machine learning approach for assessing quality from user reviews indicates that park condition and safety are the topics most commonly associated with negative park scores in areas with higher proportions of Black and Hispanic residents, disadvantaged SES groups, children, and those with disabilities (Fig. 5a). Taking advantage of these findings for future park planning in Philadelphia and elsewhere is likely to lead to an improvement in the perception of park quality for those who need it most.

Our findings suggest city officials and urban forestry managers should consider investing in improving park condition by ensuring regular maintenance and cleanliness, including mowing of grasses, trash disposal, trimming trees and shrubs, removing safety hazards, and repairing damaged infrastructure such as playground equipment, benches and seating areas. Landscaping features such as planting of flowers, improving walking paths, drinking fountains, restrooms, picnic spots and sport facilities are also crucial especially in lower income areas which correlate strongly with mentions of condition in negative park reviews. Physical safety is another area in need of significant improvements to enhance perceived park quality. However, the lack of strong correlation between reported crimes and perceived physical safety (Fig. 6) indicates that mentions of safety in Google reviews are more reliant on individuals’ perceived feelings of safety rather than the objective incidence of crime in a park. Since an increased number of police-reported crimes within a park does not lead to a strong change in park users' feelings of safety, an increased police presence is unlikely to improve perceived safety. Instead, park safety may only improve with more systemic changes to the major challenges endemic to certain groups in this region. One example is the opioid epidemic which led to 1046 fatal overdoses in 2021 in Philadelphia^34^, as many common words in the safety topic are drug related (addict, dealer, needle, etc.) (Supplementary Information Table 1). Additionally, many users mentioning dangerous and unsafe conditions in their negative reviews also cite trash, broken park equipment, and lack of lighting. This provides further evidence of the need for investment into cleaning and maintaining park infrastructure. Our results identify 17 parks (Table 1) that are ideal candidates for reducing these inequalities. It is immensely important, however, to combine any quality improvement efforts and investments with appropriate measures (e.g., rent control, anti-displacement policies, and input from community members) to prevent green gentrification in Philadelphia.

**Table 1:** 17 parks that are ideal candidates for reducing inequality due to having scores lower than 4 stars and being located in areas with high populations of demographics who have access to lower quality parks.

Park name	Park score	Planning districts	Dominant topic
Hope Park	3.2	North	Safety
Barrett Playground	3.3	Upper North	Built Amenities
McPherson Square	3.4	North	Safety
Hissey Playground	3.6	North	Safety
Morris Estate Recreation Center	3.7	Upper North	Condition
Norman Ellis Playground	3.7	West	Built Amenities
Ziehler Playground	3.8	Upper North	Built Amenities
Scanlon Playground	3.8	North	Condition
Conestoga Park	3.8	West Park	Condition
Baker Playground	3.8	West	Safety
Nelson Playground	3.8	Lower North	Safety
Olney Recreation Center	3.9	Upper North	Recreation
Houseman Playground	3.9	Lower Northeast	Recreation
McVeigh Recreation Center	3.9	North	Safety
Harrowgate Park	3.9	River Wards	Safety
Mullin Playground	3.9	North Delaware	Condition
Deni Playground	3.9	Lower Northeast	Built Amenities

The unequal patterns in park perception revealed in this study also highlight several public health concerns. For example, those in disadvantaged SES groups have lower access to health care and nutritious foods, and higher incidence of psychological distress and depression than those in advantaged SES groups^35^. Thus, individuals within disadvantaged SES groups are in greater need of public spaces for physical activity and connecting with nature which are only accessible when parks are of high quality^36–39^. Lower perceived park quality for children under 9 raises a concern for childhood health and development. Research has shown that increased park facilities benefit the physical health of children and that nature-based play in childhood promotes physical health, wellbeing, and social competence^40,41^.

It is important to note that Google Maps reviews were used in this study due to the ubiquitous use of the platform as evidenced by the high volume of reviews left for Philadelphia parks (143,913 between 2011 and 2022). However, while Google Maps is a highly accessible platform due to its free availability, there are still groups who are less likely or unable to use the platform such as those without mobile phones or internet access^42^. The general use of social media data in equity studies also creates some limitations when compared to traditional methods such as surveys. For example, Google Maps does not provide a user’s demographic information. This study attempts to overcome this limitation by linking park reviews to the demographic makeup of a census tracts to which the park is accessible. One limitation of Google Maps text-based reviews is its open-ended nature, only prompting users to “Share details of your own experience with this place”. This means that users leave reviews with varying amounts of information and relevance to the conditions of the park they are reviewing, leading to some reviews that provide a lot of useful information about park conditions and many that do not, introducing noise to the dataset^43^. We were able to reduce the influence of reviews with lower relevance to park conditions by filtering out reviews based on low letter counts and quantifying park topics through topic modeling with a set of predefined keywords that are associated with park condition (see “Methods”). Despite these limitations, the anonymity associated with Google reviews allow users to share their candid and unrestrained opinion about their park experiences, thus, providing other users and park managers valuable information for decision making while signaling areas of improvement.

Philadelphia has an extensive park system serving its diverse neighborhoods. Yet the public perception of parks based on Google Maps reviews is significantly lower in areas with higher populations of Black and Hispanic residents, disadvantaged SES groups, children, and people with disabilities. These are precisely the demographic groups who should have safe access to high-quality UGS, if we were to meet the SDG 11.7 target by 2030^7^. These findings not only reveal important environmental justice concerns but show gaps in interactional, distributive and spatial justice. Our study offers empirical evidence for undoing legacies of inequality in park quality by centering the voices and experiences of underserved communities and their lack of access to highly-rated parks and their benefits. We argue that park planners must go beyond boosting aesthetics and natural amenities in parks within White and higher educated tracts, by refocusing attention on diverse user concerns and their pressing needs for improved and quality park systems that can enhance health, safety, and overall wellbeing especially in marginalized and underserved neighborhoods.

## Methods

### Origin and destination (OD) cost matrix analysis

We used an origin and destination (OD) cost matrix^32^ analysis to determine which individual parks are accessible to different census tracts and the demographic populations living within them. In the OD cost matrix, the origins are every residential area within each census tract as derived from Philadelphia zoning data and the destinations are every park with sufficient social media data (n = 285). Residential areas are considered as origins so that only distances from where individuals are living within a census tract are considered. We further refined the park destinations to estimate park entrances—areas where park boundaries connect to road networks. A road network was obtained from the “Street Centerlines” dataset^44^. Freeways and Highways were removed from the road network dataset to only include walkable roads in the analysis. Connections were measured as the distance in meters along the walkable road network between every origin and every destination in the dataset. We assume that parks are being used and reviewed by those living near a park. Only parks within 800 m of an origin were considered as accessible, based on the commonly cited threshold for the maximum walking distance to a park^45–47^. The number of parks accessible to each census tract ranged from 1 to 22 (Supplementary Information Fig. 4).

### Park perception data from Google Maps

Park perception data were obtained from the Google Maps review page URLs for each park listed in Philadelphia Parks and Recreation’s “PPR Properties'' dataset^48^. Google Maps reviews have been effectively utilized in prior studies to assess the user perception of a number of different physical locations including restaurants, airports, and parks^49–51^. On June 8, 2022, we scraped Google Maps review data (n = 143,913) posted between 2011 and 2022 with Python using the review page URLs for each park listed in Philadelphia Parks and Recreation’s “PPR Properties'' dataset^48^. To clean the reviews for text processing, we (i) autocorrected words, (ii) removed numbers, capitalization, special characters, and common stop words, and (iii) lemmatized words to return them to their base forms (though we continue to refer to these resulting unigrams as ‘words’ above and below). An additional list of commonly used words was also removed from the reviews to reduce the high influence of very frequent words which had little bearing on the meaningful content of a review, such as ‘from’, ‘museum’, ‘translate’, ‘original’, ‘place’, ‘park’, ‘great’, ‘nice’, and ‘kid’. Altogether, this preprocessing resulted in our retaining 10,999 unique words across the 69,686 reviews. We excluded parks with fewer than 10 reviews reducing the number of parks in our dataset from 514 to 285.

Extracted data include the park name, number of reviews for the park, average park score of all the user scores for a park (with 5 stars being the highest quality and 1 being the lowest), the individual’s user score, and the text left by users describing their experience with the park. The park score of a census tract is derived from the average of all individual user scores that are accessible to the tract, measured as being within 800 m of the residential area of a tract. Park scores and demographic populations were considered high if they fell within the upper 75th percentile or low if they fell within the lower 25th percentile of their respective datasets. We then used bivariate mapping to identify where high/low park scores are associated with high/low populations of demographic groups, so as to identify areas where distributive injustice is occurring (Fig. 1). The relationship between the demographics and park score of a census tract was measured using Spearman’s correlation coefficients and p-values (Fig. 2).

### Semi-supervised topic modeling

To condense information from the large quantity of textual content contained in our preprocessed reviews, the primary topics commonly associated with urban parks were identified through a review of urban park quality measurement tools. Between 13 available park quality assessment tools, the most common qualities or characteristics were safety, built amenities, accessibility, recreation, natural amenities, condition, size, and aesthetics^27,31,52–62^. We further categorized Google text-based reviews into the eight previously mentioned park characteristics or topics using anchor word-assisted semi-supervised topic modeling in order to determine what drives users’ perceptions of a park quality (Supplementary Information Table 2). We selected the anchor words to train our model to predict the probability of these eight topics based on the 13 referenced park quality assessment tools. For this particular step, we excluded reviews with three letters or less, reducing the sample size from 143,913 to 69,686.

Anchor words associated with these topics were used in the development of a semi-supervised topic model of park reviews. Topic modeling is a method of identifying latent or hidden topics from text. Consistent with recent advances in topic modeling^63–65^, we identified ten frequently used keywords for each topic through a combination of park quality literature review, manual reading of a subset of all park reviews, and exploratory unsupervised topic modeling using Latent Dirichlet Allocation (LDA). These keywords were used as anchors in the semi-supervised topic modeling (Supplementary Information Table 1). Anchored Correlation Explanation (CorEx)^62^ was used as the primary topic model for estimation. Anchored CorEx is a semi-supervised topic model that uses an information-theoretic approach to identify latent topics. CorEx has shown comparable results to other topic models such as LDA^66^ and was used in this study due to (i) its ability in identifying underrepresented topics and (ii) its recent successes in modeling topics from social media data in similar environmental and health domains^67,68^. Further details about the data cleaning process, hyperparameters for the CorEx model, and interpretation of the model results can be found in the Supplementary Information section.

The dominant topic per review was used to understand what topics a review was most likely to represent and was determined by the maximum topic probability in a review which is consistent with past topic modeling applications^66,69,70^. Dominant topics were calculated at the park level by averaging topic probabilities across all reviews for a given park and selecting the topic associated with the maximum probability. To scale this to the census tract level, topic probabilities per park were averaged for all parks accessible to a census tract as based upon an 800 m distance threshold. We measured the relationship between topic probabilities and park scores through Spearman’s correlation coefficients and p-values for each topic and park score at the tract-level (Supplementary Information Fig. 2). Spearman’s correlation coefficients were also calculated between each of the topics and the 21 socioeconomic variables at the tract-level (Supplementary Information Tables 3 and 4).

### High-resolution aerial data for observed park characteristics

Observed park characteristics were derived from remotely sensed aerial and satellite imagery and GIS data from OpenDataPhilly to quantify the park topics utilized in our topic model. Our classification of park-based land cover via high-resolution remote sensed data is novel, but aligns well with past studies classifying similar urban spaces^71^. Remote sensing imagery was transformed into discrete values by classifying image pixels as trees, grass, built land, or water. To obtain high-resolution data on the landscape composition of each park, aerial images from the National Agricultural Imagery Program (NAIP; spatial resolution: 1 m) and Sentinel-2 images (spatial resolution: 10 m) were classified using a random forest (RF) classifier in Google Earth Engine (GEE), a cloud-based platform for geospatial analysis^72^^.^ NAIP images were captured between June 11th, 2017 and June 13th, 2017, whereas Sentinel-2 images were acquired between June 1st, 2017 and September 30th, 2017 during the leaf-on period. The usage of imagery collected while the trees have foliage allows the full extent tree canopy to be classified; however, some built, grass, and water features will be obstructed.

The RF model was trained using 8,961 reference points digitally collected from visual assessment of the NAIP imagery, of which 70% were used to train the model and 30% used to test accuracy. The model achieved a high accuracy of ~ 93% (Supplementary Information Table 5). The high resolution of NAIP often leads to the misclassification of single pixels creating a speckle effect. The inclusion of Sentinel-2 images in the classification provides spectral information surrounding the 1 m pixel while retaining a classified map with a high 1 m spatial resolution. Without the inclusion of Sentinel-2 images in the classifiers, overall accuracy decreased from ~ 93 to ~ 85%. Further information about the remote sensing images that were used in the RF model and the hyperparameters for the RF model can be found in Supplementary Information.

For each park, the area of each of the four land cover classes was divided by the total park area to measure the relative coverage of each land cover class (Supplementary Information Fig. 5). Tree coverage was used as an observed measure for the perception of natural amenities, whereas water coverage was used as a measure for natural amenities and aesthetics. Built coverage was used as a measure for built amenities, and grass coverage was used as a measure for recreation. Additional observed variables were obtained from the Philadelphia Police Department and Philadelphia Parks and Recreation to compare to perceived topics including violent crimes, narcotic crimes, sports facilities, park areas (Supplementary Information Fig. 3). A Spearman’s correlation matrix was calculated to quantify the relationship between the observed measurements and perceived topics within a park (p-values in Supplementary Information Table 6).

## Supplementary Information

Supplementary Information.

## Data Availability

The land cover classified datasets^73^ for Philadelphia are available at https://zenodo.org/record/7604104. The GIS data used to delineate park boundaries (https://www.opendataphilly.org/dataset/ppr-properties), streets (https://www.opendataphilly.org/dataset/street-centerlines), residential areas (https://www.opendataphilly.org/dataset/zoning-base-districts), incidence of crimes (https://www.opendataphilly.org/dataset/crime-incidents), and park facilities (https://www.opendataphilly.org/dataset/city-facilities-master-facilities-database) are publicly available at OpenDataPhilly.org.

## Code availability

The land cover classification was performed in the geospatial platform Google Earth Engine and code is available at https://code.earthengine.google.com/32d3a77e70955a6279ec22233778bd8f. Topic modeling, data analysis and plots were run in Python and data is available at https://github.com/mattswalter/Philadelphia_Park_Scores/tree/main.
