# Supplementary material for: Social media analysis reveals environmental injustices in Philadelphia urban parks
Source: Sci Rep. 2023 Aug 3;13:12571. doi: 10.1038/s41598-023-39579-4 (PMC10400556; doi:10.1038/s41598-023-39579-4)
Supplement: Supplementary file 1 — Supplementary Information. [file 41598_2023_39579_MOESM1_ESM.docx]

**Social Media Analysis Reveals Environmental Injustices in Philadelphia Urban Parks**

Matthew Walter, Benjamin E. Bagozzi, Idowu Ajibade, Pinki Mondal

Matthew Walter

Email: mswalter@udel.edu

**This PDF file includes:**

Supporting text

Figures S1 to S5

Tables S1 to S6

SI References

Supporting Information Text

**CorEx model input and parameters.**

To remove noise from the vocabulary set, words appearing in less than five documents and more than 50% of documents were removed leaving 4,542 unique words that were used as model input. The number of hidden topics was set to eight to capture our eight defined park characteristics, and the strength of the anchor words was set to 12 to steer the model towards our pre-selected topics without overfitting any topic to its set of anchor words. As a review-level measure of topical association, the probability of a given topic (y)'s occurrence in a particular review, as based upon that review's observed word type grouping x, is defined as log p(y|x). Using the corresponding log p(y|x) estimates from the CorEx model described above, eight topic indexed versions of this associational quantity are recovered for each relevant review and are standardized using z-scores. Reviews without at least one topic containing a z-score value greater than 0.5 were removed. The purpose of doing so was to avoid making inferences about associations between topics and review- or park-level attributes in instances where no single dominant topic is evident [1]. A threshold of 0.5 was selected by manually assessing the minimum z-scores in which a topic was still observable within a review. This threshold was also found to remove large amounts of reviews which didn’t fall into any topic being categorized as a single topic. This threshold reduced the number of reviews from 69,686 to 29,738

**Random Forest (RF) Model and Remote Sensing Imagery.**

RF is a supervised machine learning algorithm widely used in land cover classification due to their effectiveness, robustness, and implementation into GEE [2,3,4,5]. RF uses an ensemble of randomly sampled decision trees, then uses majority voting across all trees to make a prediction [6]. We set the number of trees in our model to 100, as more than 128 yields little to no performance improvement [7]. NAIP images include red, green, blue, and near-infrared (NIR) bands collected during the summer of 2017. These images were mosaiced and clipped to the City of Philadelphia Boundary. To increase classification accuracy, an additional image band for vegetation health (Normalized Difference Vegetation Index (NDVI)) was calculated from the NAIP imagery using the following formula [8,9,10,11]:

NDVI = (NIR - red)/(NIR + red)

Image classification using NAIP data has the potential to suffer due spectral and temporal limitations^8^. To address this concern and improve classification accuracy, we incorporated additional remote sensing data from Sentinel-2 [12]. A median composite of 10-m and 20-m Sentinel-2, Level-1C data was calculated using cloud-masked imagery collected over the summer (June-September) of 2017. The following bands were included in the RF model: blue, green, red, red edge 1, red edge 2, red edge 3, NIR, and shortwave infrared 1. Sentinel-2 data were combined with the NAIP data using the ee.Image.addBands function in GEE. The ee.Image.addBands function retains and rescales to a 1-m resolution across all NAIP and Sentinel-2 bands by outputting a new image with the same metadata and footprint as the first image included in the function [13].

Using the RF model, the NAIP and Sentinel-2 composite was classified into four broad land cover features typically found in urban parks: trees, grass, built, and water. To reduce the speckle effect, a 3x3 square kernel was used to smooth the classified image by calculating the mode of a pixel's neighbors. Measures of accuracy including overall accuracy and per class user’s and producer’s accuracy of the RF classifier were calculated and are presented in Table S5 [14].


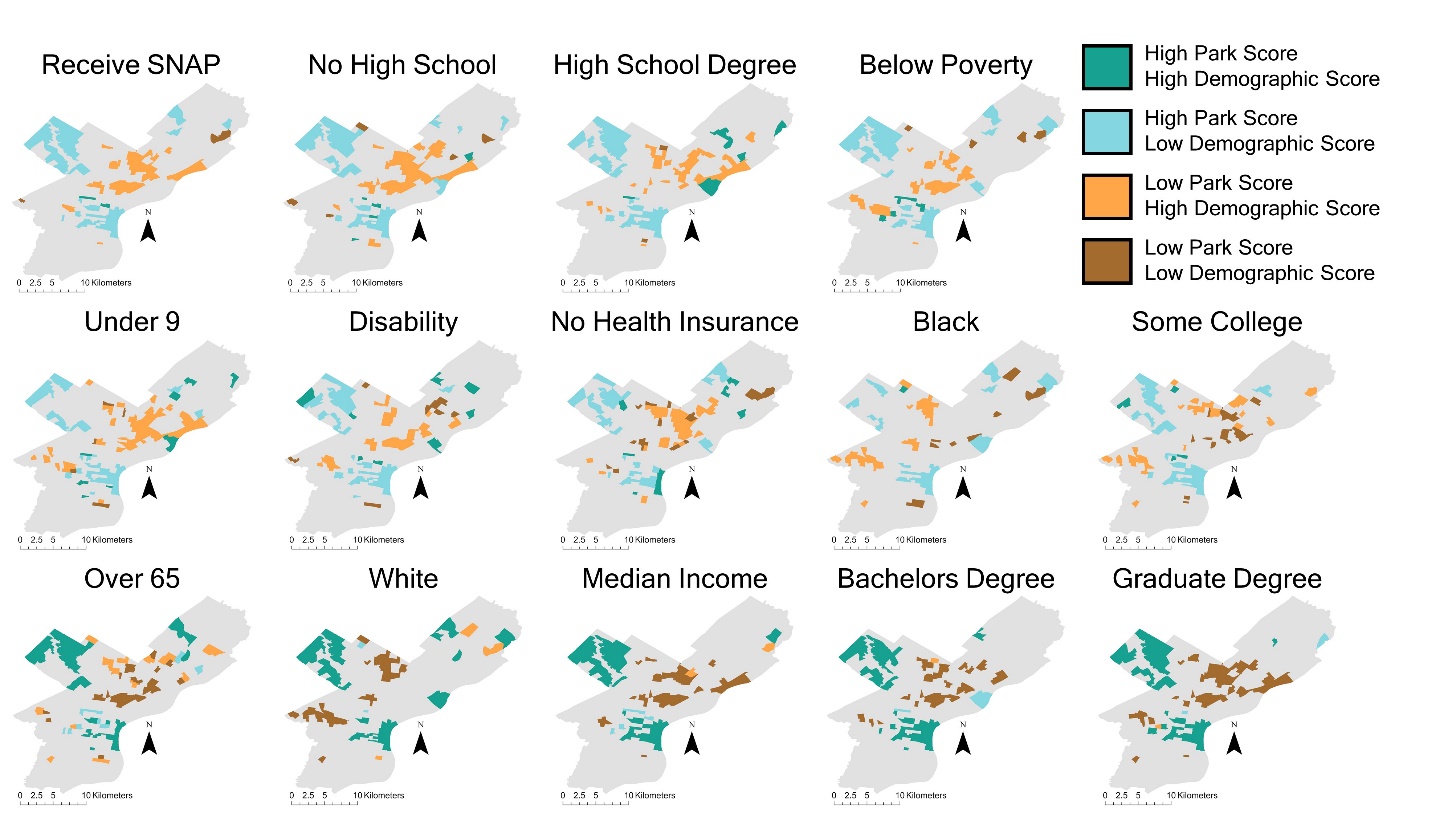


**Figure S1.** Relationships between all significant demographics (education, income, race, disability, and age) and their park score at the census tract level, measured as the average Google Map score of all parks within 800-m of a tract’s residential areas. These bivariate maps can be used to identify areas in need of park improvements due to having low park scores and high concentrations of certain demographic groups.


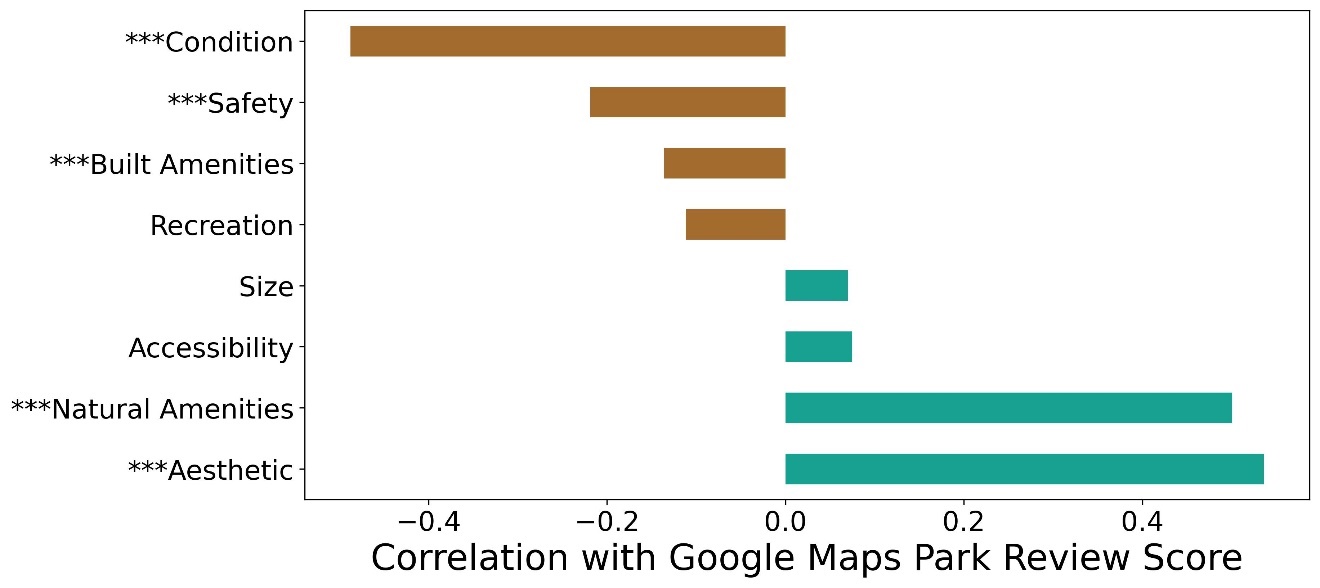


Figure S2. Correlation coefficients between the distribution of perceived topic probabilities and the average score of a park.

**
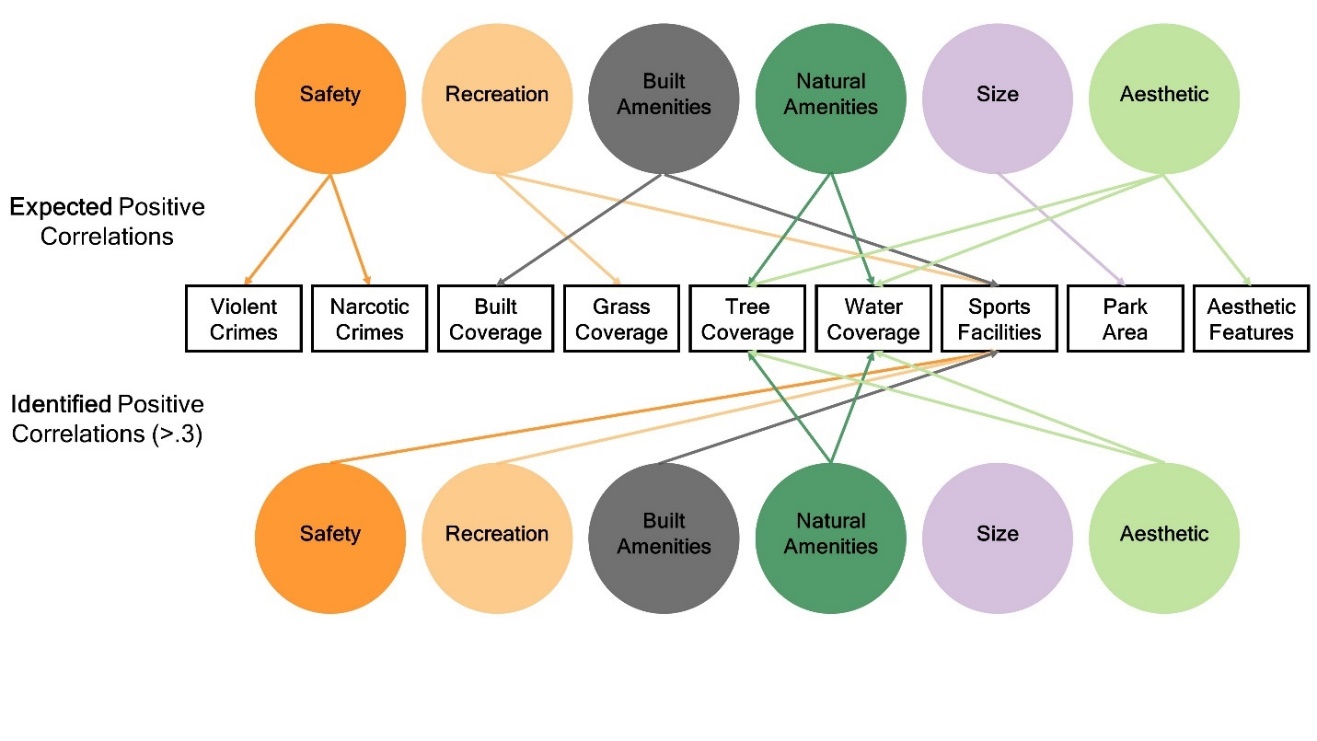
**

Figure S3. Comparison of perceived topics and their expected associations to observed physical data variables.


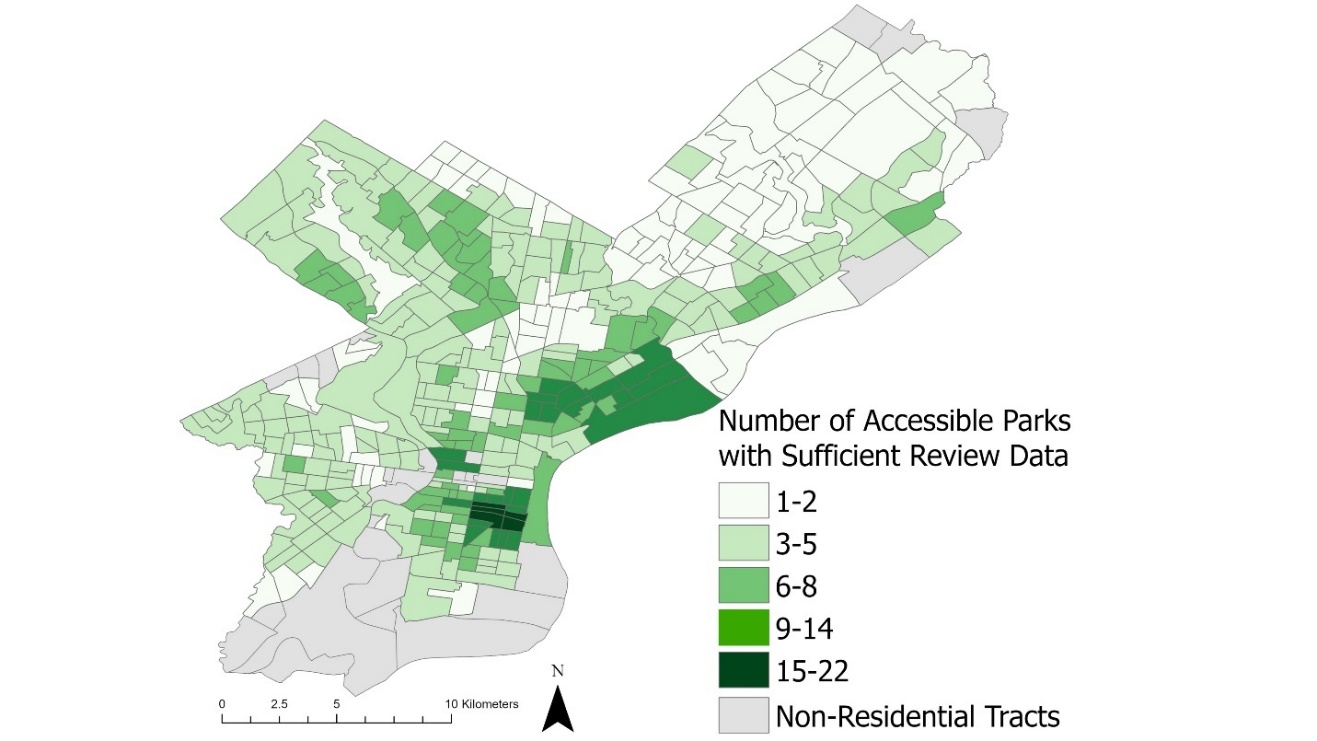


Figure S4. The number of parks that have sufficient Google Maps reviews (10 or more) and are accessible (within 800 m) to each census tract.


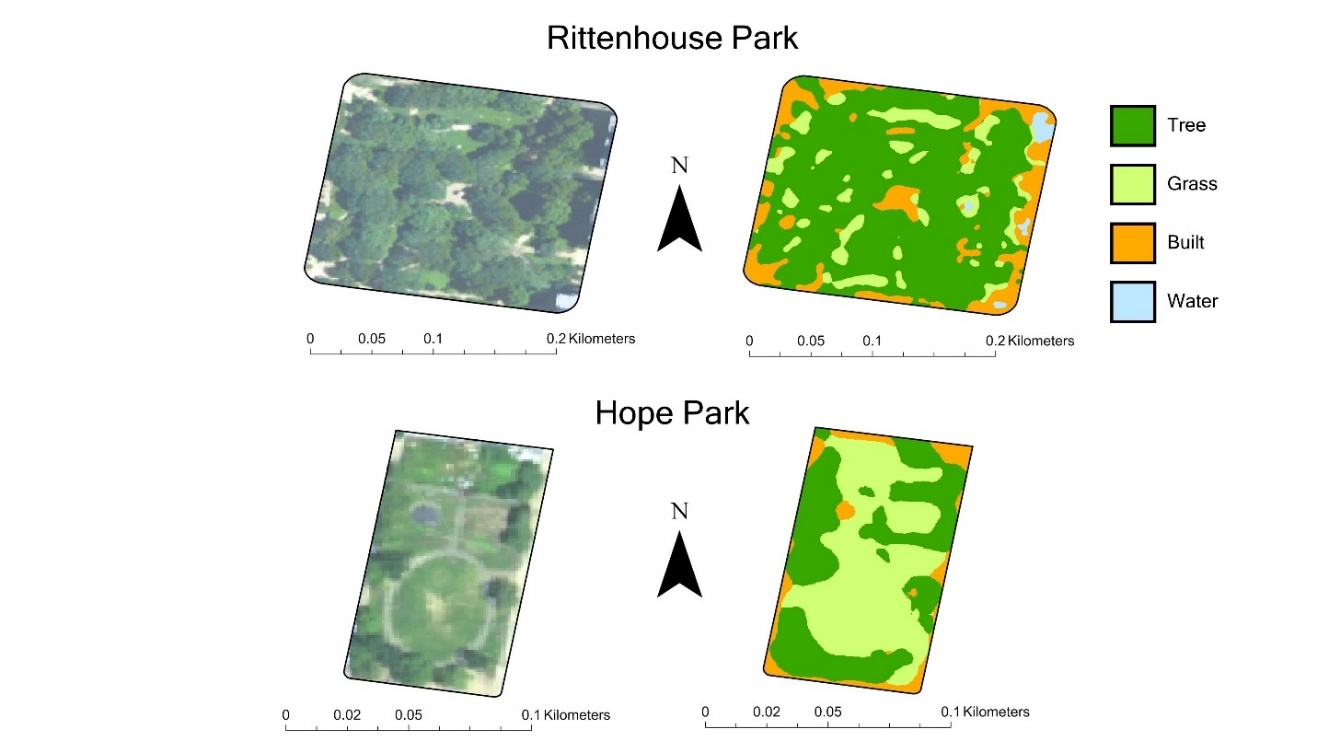


Figure S5. Landcover classification showing tree coverage, grass coverage, built coverage, and water coverage for two parks.

Table S1. Top 10 words associated with each topic in the semi-supervised CorEx model when including and excluding anchor words.

| **Topic** | **Top 10 (with anchor words)** | **Top 10 (without anchor words)** |
| --- | --- | --- |
| Safety | safe, drug, smoking, security, dangerous, needle, hurt, crime, creepy, violence | people, felt, addict, weed, dealer, careful, feel, police, emailed, infested |
| Recreation | walk, basketball, hike, baseball, tennis, sport, recreation, soccer, workout, swim | bike, field, dog, run, jog, ride, football, center, league, game |
| Built Amenities | playground, light, fountain, bench, court, bathroom, path, facility, table, shelter | play, equipment, water, pool, area, sit, plenty, animal, carousel, need |
| Natural Amenities | nature, garden, tree, river, environment, flower, blossom, plant, pond, landscape | cheery, beer, Japanese, Delaware, enjoy, Schuylkill, house, bloom, lovely, spring |
| Accessibility | open, close, expensive, hidden, entrance, road, access, cost, affordable, connected | gem, food, space, closed, exhibit, covid, price, easy, wide, ticket |
| Condition | clean, dirty, broken, update, maintenance, condition, fix, upkeep, filthy, mess | friendly, kept, staff, trash, glass, maintained, helpful, restroom, care, healthy |
| Size | big, crowded, large, busy, size, spacious, tiny, overcrowded, cat, acre | small, lot, bit, weekend, time, day, monkey, weekend, cat, time |
| Aesthetic | beautiful, view, peaceful, pretty, atmosphere, scenery, calming, loud, charm, city | city, trail, skyline, serene, relaxing, fun, wedding, quiet, bridge, hermoso |

Table S2. Description of the eight park characteristics input into the topic model.

| Park Characteristic | Description | References |
| --- | --- | --- |
| Safety | Perceived feeling of safety or objective likelihood of crime which are influenced by the frequency of violent crimes, lighting, emergency infrastructure, and security within and surrounding a park | Bedimo-Rung et al., 2005 [15]; Byrne et al., 2005 [16]; Kaczynski et al., 2012 [17]; Rigolon & Németh, 2018 [18] |
| Built Amenities | Human-built facilities and infrastructure within parks such as signs, restrooms, benches, basketball courts, and playgrounds | Saelens et al., 2006 [19] |
| Accessibility | Availability of park space, equitable distribution of park space, the ability of an individual to get to a park, and the ability of individuals to move around easily inside a park, with metrics including the need for reservations, proximity of public transportation, and traffic | Bedimo-Rung et al., 2005 [15]; Kaczynski et al., 2012 [17] |
| Recreation | Opportunity to participate in physically active and sedentary activities including walking, sports, viewing scenery, and family gatherings | Bedimo-Rung et al., 2005 [15] |
| Natural Amenities | The presence of natural/environmental features within a park such as trees, wetlands, and rivers | Byrne et al., 2005 [16] |
| Condition | The maintenance level of all components of a park, including indicators such as litter, graffiti, overgrown landscaping, and damaged infrastructure | Byrne et al., 2005 [16]; Cavnar et al., 2004 [20] |
| Size | Relative area that an individual park covers compared to other parks within a region | Lee et al., 2005 (21); Rigolon & Németh, 2018 [18] |
| Aesthetics | The perceived attractiveness of a park’s design elements which can be influenced by a park’s layout and visual scenery | Bedimo-Rung et al., 2005 [15]; Rigolon & Németh, 2018 [18] |

| **Table S3.** Spearman’s correlation coefficients between the demographics of a census tract and the topic probabilities of parks accessible to a tract when only including negative reviews (1-3 stars). ***p < 0.01, **p < 0.05, *p < 0.1 | | | | | | | | |
| --- | --- | --- | --- | --- | --- | --- | --- | --- |
|  | **Safety** | **Recreation** | **Built Amenities** | **Natural Amenities** | **Accessibility** | **Condition** | **Size** | **Aesthetic** |
| **Receive SNAP** | 0.28*** | -0.04 | 0.05 | -0.27*** | -0.21*** | 0.44*** | 0.02 | -0.21*** |
| **No High School Degree** | 0.24*** | -0.02 | 0.05 | -0.22*** | -0.17*** | 0.37*** | 0.01 | -0.27*** |
| **High School Degree** | 0.22*** | 0.03 | 0.11** | -0.24*** | -0.24*** | 0.34*** | -0.03 | -0.29*** |
| **Below Poverty** | 0.22*** | -0.08 | -0.01 | -0.19*** | -0.15*** | 0.36*** | 0.05 | -0.12** |
| **Under 9** | 0.23*** | -0.09* | 0.01 | -0.15*** | -0.23*** | 0.31*** | -0.02 | -0.25*** |
| **Disability** | 0.21*** | -0.04 | 0.03 | -0.22*** | -0.07 | 0.26*** | -0.03 | -0.19*** |
| **No Health Insurance** | 0.11** | -0.02 | 0.02 | -0.14*** | -0.21*** | 0.32*** | -0.02 | -0.16*** |
| **Black** | 0.01 | -0.02 | 0.09* | -0.25*** | -0.32*** | 0.23*** | 0.01 | -0.14*** |
| **Some College** | 0.00 | 0.01 | 0.13** | -0.24*** | -0.35*** | 0.28*** | -0.08 | -0.24*** |
| **Unemployed** | 0.03 | 0.00 | 0.06 | -0.15** | -0.21*** | 0.23*** | 0.07 | -0.17*** |
| **Limited English** | 0.20*** | 0.01 | 0.03 | 0.09* | -0.04 | 0.19*** | 0.16*** | -0.12** |
| **Hispanic** | 0.24*** | -0.02 | -0.07 | 0.02 | 0.09* | 0.11** | 0.09* | -0.07 |
| **Female** | 0.02 | 0.02 | 0.06 | -0.04 | -0.06 | 0.01 | 0.02 | 0.03 |
| **Male** | -0.02 | -0.02 | -0.06 | 0.04 | 0.06 | -0.01 | -0.02 | -0.03 |
| **Median Age** | -0.06 | 0.01 | 0.01 | -0.04 | -0.02 | -0.10** | -0.07 | -0.01 |
| **Asian** | -0.04 | 0.05 | 0.00 | 0.30*** | 0.11** | -0.08 | 0.23*** | 0.05 |
| **Over 65** | -0.09* | 0.03 | 0.01 | 0.16*** | 0.08 | -0.24*** | -0.04 | 0.11 |
| **White** | -0.09* | 0.09* | -0.11** | 0.27*** | 0.35*** | -0.35*** | -0.05 | 0.20*** |
| **Median Income** | -0.24*** | 0.06 | -0.03 | 0.23*** | 0.21*** | -0.42*** | -0.03 | 0.19*** |
| **Bachelor's Degree** | -0.25*** | 0.06 | -0.10* | 0.31*** | 0.26*** | -0.38*** | -0.02 | 0.29*** |
| **Graduate Degree** | -0.27*** | 0.01 | -0.14*** | 0.33*** | 0.26*** | -0.39*** | 0.04 | 0.28*** |

| **Table S4.** Spearman’s correlation coefficients between the demographics of a census tract and the topic probabilities of parks accessible to a tract when only including positive reviews (4-5 stars). ***p < 0.01, **p < 0.05, *p < 0.1 | | | | | | | | |
| --- | --- | --- | --- | --- | --- | --- | --- | --- |
|  | **Safety** | **Recreation** | **Built Amenities** | **Natural Amenities** | **Accessibility** | **Condition** | **Size** | **Aesthetic** |
| **Receive SNAP** | 0.23*** | 0.11** | -0.07 | -0.33*** | -0.18*** | 0.30*** | -0.14** | -0.26*** |
| **No High School Degree** | 0.22*** | 0.08 | 0.06 | -0.28*** | -0.16*** | 0.29*** | -0.09* | -0.27*** |
| **High School Degree** | 0.15*** | 0.16*** | 0.03 | -0.28*** | -0.18*** | 0.26*** | -0.18*** | -0.35*** |
| **Below Poverty** | 0.20*** | 0.00 | -0.09* | -0.22*** | -0.26*** | 0.20*** | -0.06 | -0.10** |
| **Under 9** | 0.21*** | 0.07 | 0.00 | -0.30*** | -0.11** | 0.34*** | -0.11** | -0.24*** |
| **Disability** | 0.17*** | 0.11** | -0.05 | -0.27*** | -0.11** | 0.26*** | -0.25*** | -0.18*** |
| **No Health Insurance** | 0.18*** | 0.14 | 0.01 | -0.17*** | -0.07 | 0.18*** | -0.03 | -0.24*** |
| **Black** | -0.02 | 0.30*** | -0.15*** | -0.22*** | -0.34*** | 0.02 | -0.22*** | -0.13** |
| **Some College** | 0.08 | 0.28*** | 0.08 | -0.27*** | -0.20*** | 0.19*** | -0.14*** | -0.30*** |
| **Unemployed** | 0.15*** | 0.17*** | -0.03 | -0.10* | -0.14*** | 0.16*** | -0.14*** | -0.16*** |
| **Limited English** | 0.16*** | -0.03 | 0.12** | -0.10** | 0.11** | 0.14*** | 0.08 | -0.15*** |
| **Hispanic** | 0.18*** | -0.06 | 0.06 | -0.10** | 0.10* | 0.17*** | -0.03 | -0.11** |
| **Female** | 0.00 | 0.04 | -0.11** | -0.04 | -0.12** | -0.04 | -0.10* | 0.06 |
| **Male** | 0.00 | -0.04 | 0.11** | 0.04 | 0.12** | 0.04 | 0.10* | -0.06 |
| **Median Age** | -0.15*** | 0.09* | 0.08 | 0.03 | 0.10* | -0.04 | -0.14*** | -0.01 |
| **Asian** | -0.04 | -0.09* | 0.14*** | 0.26*** | 0.12 | -0.11** | 0.20*** | 0.17*** |
| **Over 65** | -0.18*** | -0.01 | -0.01 | 0.21*** | 0.16*** | -0.17*** | 0.07 | 0.16*** |
| **White** | -0.06 | -0.25*** | 0.14*** | 0.27*** | 0.31*** | -0.11** | 0.19*** | 0.20*** |
| **Median Income** | -0.19*** | -0.11** | 0.07 | 0.29*** | 0.27*** | -0.23*** | 0.10** | 0.21*** |
| **Bachelor's Degree** | -0.20*** | -0.15*** | 0.05 | 0.32*** | 0.25*** | -0.27*** | 0.18*** | 0.31*** |
| **Graduate Degree** | -0.23*** | -0.15*** | -0.03 | 0.37*** | 0.22*** | -0.32*** | 0.15*** | 0.36*** |

**Table S5.** Overall classification accuracy, user’s accuracy, and producer’s accuracy for the four land cover classes included in the RF classification.

| **Land Cover Class** | **Overall Accuracy** | **User’s Accuracy** | **Producer’s Accuracy** |
| --- | --- | --- | --- |
|  | 93% |  |  |
| Tree |  | 89.73% | 93.90% |
| Grass |  | 93.41% | 88.21% |
| Built |  | 98.28% | 97.44% |
| Water |  | 93.56% | 98.95% |

|  | **Narcotic Crimes** | **Violent Crimes** | **Built Coverage** | **Grass Coverage** | **Tree Coverage** | **Water Coverage** | **Sports Facilities** | **Park Area** | **Aesthetic Features** |
| --- | --- | --- | --- | --- | --- | --- | --- | --- | --- |
| **Safety** | 0.18*** | 0.21*** | 0.07 | 0.20*** | -0.18*** | -0.21*** | 0.338*** | -0.04 | -0.15*** |
| **Recreation** | 0.00 | 0.06 | 0.06 | 0.24*** | -0.24*** | -0.05 | 0.42*** | 0.14** | -0.09 |
| **Built Amenities** | -0.02 | 0.00 | 0.29*** | 0.22*** | -0.37*** | -0.28*** | 0.51*** | -0.12** | -0.18*** |
| **Natural Amenities** | -0.17*** | -0.19*** | -0.34*** | -0.15*** | 0.43*** | 0.35*** | -0.35*** | 0.16*** | 0.15** |
| **Accessibility** | -0.30*** | -0.30*** | -0.14** | 0.01 | 0.08 | 0.16*** | -0.06 | 0.15 | -0.01 |
| **Condition** | 0.13** | 0.19*** | 0.09 | 0.16*** | -0.24*** | -0.24*** | 0.29*** | -0.06 | -0.19*** |
| **Size** | -0.16*** | -0.16*** | -0.11* | 0.06 | 0.08 | 0.03 | 0.02 | 0.04 | 0.07 |
| **Aesthetic** | -0.06 | -0.08 | -0.29*** | -0.21*** | 0.44*** | 0.32*** | -0.43*** | 0.14** | 0.18*** |

**Table S6.** P-values for the spearman’s correlation between the physical properties of a park and its perceived characteristics. ***p < 0.01, **p < 0.05, *p < 0.1

**SI References**

1. Figueiredo, F. *et al.* Word co-occurrence features for text classification. *Inf. Syst.* **36**, 843–858 (2011).
2. Gislason, P. O., Benediktsson, J. A. & Sveinsson, J. R. Random Forests for land cover classification. *Pattern Recognit. Lett.* **27**, 294–300 (2006).
3. Noi Phan, T., Kuch, V. & Lehnert, L. W. Land Cover Classification using Google Earth Engine and Random Forest Classifier—The Role of Image Composition. *Remote Sens.* **12**, 2411 (2020).
4. Rodriguez-Galiano, V. F., Ghimire, B., Rogan, J., Chica-Olmo, M. & Rigol-Sanchez, J. P. An assessment of the effectiveness of a random forest classifier for land-cover classification. *ISPRS J. Photogramm. Remote Sens.* **67**, 93–104 (2012).
5. Walter, M. & Mondal, P. A Rapidly Assessed Wetland Stress Index (RAWSI) Using Landsat 8 and Sentinel-1 Radar Data. *Remote Sens.* **11**, 2549 (2019).
6. Breiman, L. Random forests. *Mach. Learn.* **45**, 5–32 (2001).
7. Oshiro, T. M., Perez, P. S. & Baranauskas, J. A. How many trees in a random forest? in *Lecture Notes in Computer Science (including subseries Lecture Notes in Artificial Intelligence and Lecture Notes in Bioinformatics)* **7376 LNAI**, 154–168 (2012).
8. Maxwell, A. E., Warner, T. A., Vanderbilt, B. C. & Ramezan, C. A. Land Cover Classification and Feature Extraction from National Agriculture Imagery Program (NAIP) Orthoimagery: A Review. *Photogramm. Eng. Remote Sensing* **83**, 737–747 (2017).
9. Rouse, J., Haas, R. H., Schell, J. A. & Deering, D. Monitoring vegetation systems in the great plains with ERTS. *NASA. Goddard Sp. Flight Cent. 3d ERTS-1 Symp., Vol. 1, Sect. A* (1974).
10. Tucker, C. J., Townshend, J. R. G. & Goff, T. E. African Land-Cover Classification Using Satellite Data. *Science (80-. ).* **227**, 369–375 (1985).
11. Townshend, J. R. G., Goff, T. E. & Tucker, C. J. Multitemporal Dimensionality of Images of Normalized Difference Vegetation Index at Continental Scales. *IEEE Trans. Geosci. Remote Sens.* **GE**-**23**, 888–895 (1985).
12. ESA. Copernicus Sentinel 2. (2022). Available at: https://sentinel.esa.int/web/sentinel/missions/sentinel-2. (Accessed: 27th January 2022)
13. GEE. ee.Image.addBands. (2022). Available at: https://developers.google.com/earth-engine/apidocs/ee-image-addbands. (Accessed: 10th November 2022)
14. Congalton, R. G. A review of assessing the accuracy of classifications of remotely sensed data. *Remote Sens. Environ.* **37**, 35–46 (1991).
15. Bedimo-Rung, A. L., Gustat, J., Tompkins, B. J., Rice, J. & Thomson, J. Development of a Direct Observation Instrument to Measure Environmental Characteristics of Parks for Physical Activity. *J. Phys. Act. Heal.* **3**, S176–S189 (2006).
16. Byrne, J., Wolch, J., Swift, J. & Ryan, C. *SAGE (Systematic Audit Of Green-Space Environments) Audit Form and Instructions*. (2005).
17. Kaczynski, A. T., Wilhelm Stanis, S. A. & Besenyi, G. M. Development and Testing of a Community Stakeholder Park Audit Tool. *Am. J. Prev. Med.* **42**, 242–249 (2012).
18. Rigolon, A. & Németh, J. A QUality INdex of Parks for Youth (QUINPY): Evaluating urban parks through geographic information systems. *Environ. Plan. B Urban Anal. City Sci.* **45**, 275–294 (2016).
19. Saelens, B. E. *et al.* Measuring Physical Environments of Parks and Playgrounds: EAPRS Instrument Development and Inter-Rater Reliability. *J. Phys. Act. Health* **3**, S190–S207 (2006).
20. Cavnar, M. M. *et al.* Evaluating the Quality of Recreation Facilities: Development of an Assessment Tool. *J. Park Recreat. Admi.* **22**, (2004).
21. Lee, R. E., Booth, K. M., Reese-Smith, J. Y., Regan, G. & Howard, H. H. The Physical Activity Resource Assessment (PARA) instrument: Evaluating features, amenities and incivilities of physical activity resources in urban neighborhoods. *Int. J. Behav. Nutr. Phys. Act.* **2**, 13 (2005).
